# Characterizing uncertainty in Community Land Model version 5 hydrological applications in the United States

**DOI:** 10.1038/s41597-023-02049-7

**Published:** 2023-04-06

**Authors:** Hongxiang Yan, Ning Sun, Hisham Eldardiry, Travis B. Thurber, Patrick M. Reed, Keyvan Malek, Rohini Gupta, Daniel Kennedy, Sean C. Swenson, Linying Wang, Dan Li, Chris R. Vernon, Casey D. Burleyson, Jennie S. Rice

**Affiliations:** 1 Pacific Northwest National Laboratory, Richland, WA USA; 2 Department of Civil and Environmental Engineering, Cornell University, Ithaca, NY USA; 3 National Center for Atmospheric Research, Boulder, CO USA; 4 Department of Earth and Environment, Boston University, Boston, MA USA

**Keywords:** Hydrology, Climate sciences

## Abstract

Land surface models such as the Community Land Model Version 5 (CLM5) are essential tools for simulating the behavior of the terrestrial system. Despite the extensive application of CLM5, limited attention has been paid to the underlying uncertainties associated with its hydrological parameters and how these uncertainties affect water resource applications. To address this long-standing issue, we use five meteorological datasets to conduct a comprehensive hydrological parameter uncertainty characterization of CLM5 over the hydroclimatic gradients of the conterminous United States. Key datasets produced from the uncertainty characterization experiment include: a benchmark dataset of CLM5 default hydrological performance, parameter sensitivities for 28 hydrological metrics, and large-ensemble outputs for CLM5 hydrological predictions. The presented datasets will assist CLM5 calibration and support broad applications, such as evaluating drought and flood vulnerabilities. The datasets can be used to identify the hydroclimatological conditions under which parametric uncertainties demonstrate substantial effects on hydrological predictions and clarify where further investigations are needed to understand how hydrological prediction uncertainties interact with other Earth system processes.

## Background & Summary

The seasonal variability of streamflow has led civilization to rely on built infrastructure, such as levees and dams, for flood control, water supply, crop production, and clean electricity^1–4^. With extreme events increasing under a changing climate, reliable hydrological predictions are key to improving strategic planning and the operation of water infrastructure^5–10^. Large-scale land surface models (LSMs) have long been essential tools for predicting future hydrology. LSMs are used in Earth-system model frameworks to link land surface processes with other, interacting processes to predict the impacts of a changing climate and evolving human systems^11–14^. Here we focus on one of the most dominantly used LSMs, the latest version of the Community Land Model (CLM), CLM5^15^. CLM5 is the land component of the Community Earth System Model, the Euro-Mediterranean Center on Climate Change coupled Earth System model^16^, and the Norwegian Earth System Model^17^. Because of the structural complexity and computationally expensive nature of CLM5, limited attention has been given to addressing uncertainties in its default hydrological parameters and how these uncertainties might impact hydrological predictions and subsequent decision-making^18–20^.

In practice, CLM5 users typically adopt the default parameter values provided by developers. These values are estimated based on limited/empirical data or calibrated deterministic values reported in the literature for a limited number of basins^21^. Moreover, prior hydrological calibration efforts for LSMs frequently only use one error metric (e.g., Nash-Sutcliffe Efficiency [NSE])^21–23^, which narrows their focus to one aspect of the flow duration curve (i.e., high flows) and can lead to significant inadvertent biases in hydrological predictions. Neglecting parameter uncertainties also can lead to biased decision-making. For example, ignoring parameter uncertainty in riverine flood prediction biases homeowners’ house-elevation decisions results, potentially resulting in higher projected economic costs^24^. Ignoring parameter uncertainty in crop yield projection under climate change biases crop insurance policies^25^. As a result, uncertainty characterization (UC) of hydrological parameters in LSM predictions is critical to informing how model parameterization influences model outcomes and applications^26^. For this work, we define UC as “*model evaluation under alternative hydrological parameterization hypotheses to explore their implications for model output uncertainty*”^27^.

To support the broad adoption of UC in CLM5 applications, we developed benchmark CLM5 hydrological datasets based on extensive UC of CLM5 hydrological parameters for 464 basins that are part of the Catchment Attributes and Meteorology for Large-sample Studies (CAMELS)^28,29^ basins over the conterminous United States (CONUS). The original CAMELS data set includes 671 headwater-type basins with minimal human influence across the CONUS. CAMELS provides basin area information from two different sources: the national geospatial fabric polygon^30^ and the United States Geological Survey Geospatial Attributes of Gages for Evaluating Streamflow version II database^31^. Following the recommendation of Addor, *et al*.^28^ not to use basins with large area discrepancies between the two sources, we identified 464 out of the 671 basins with a basin area relative difference of less than 2% as suitable for CLM5 evaluation.

**Fig. 1:**
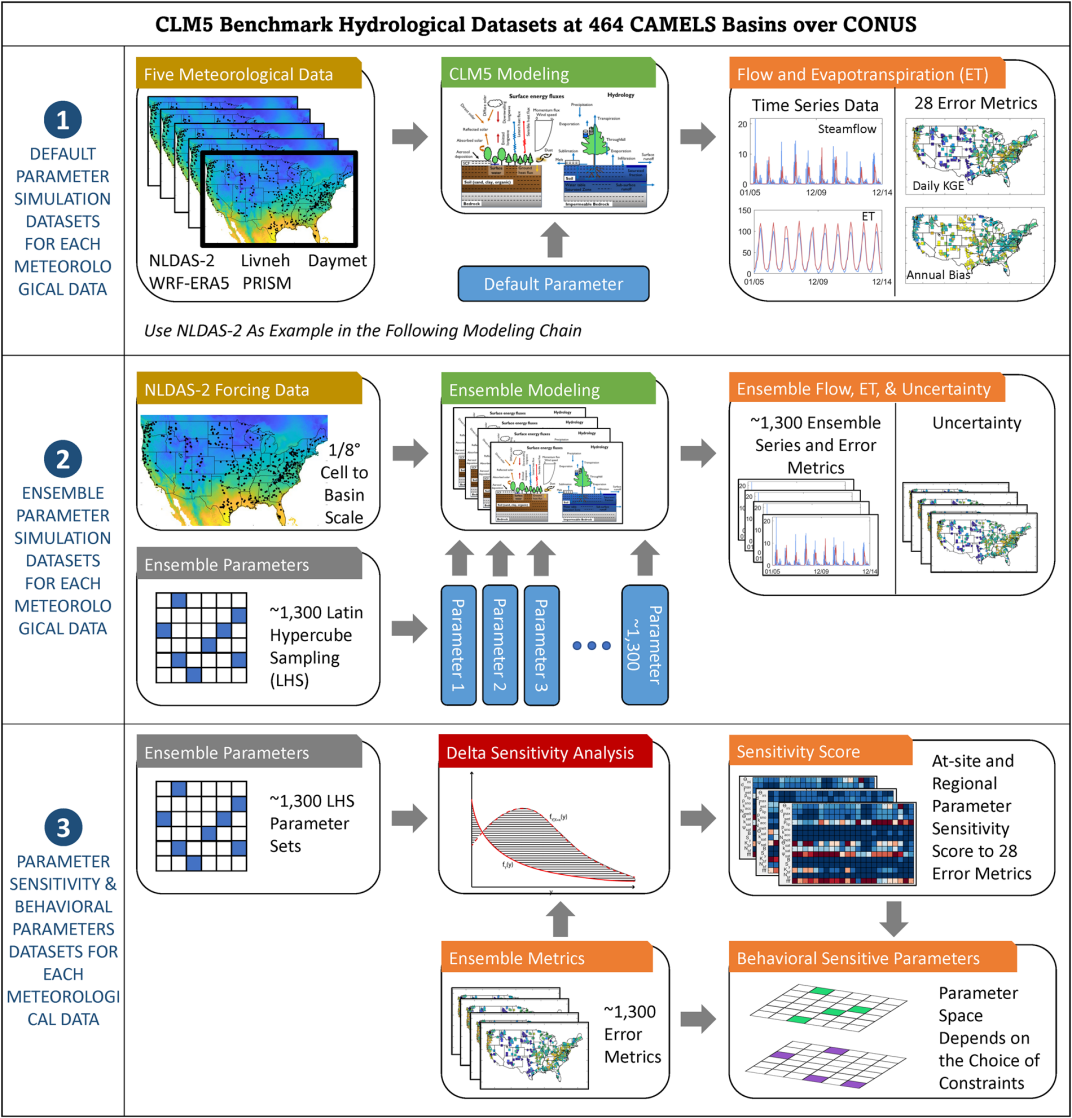
A schematic view of the CLM5 benchmark hydrological datasets. In step 2, about 1,300 ensemble parameter sets are generated using a Latin Hypercube Sampling method to produce about 1,300 ensemble time series and error metrics. The same ensemble parameters and error metrics are used in step 3 to generate at-site and regional parameter sensitivity scores as well as behavioral sensitive parameters.

Five common meteorological forcing datasets are also used to characterize the forcing data selection effects. As shown in Fig. 1, the datasets consist of three parts for each meteorological data type:

1. Performance of CLM5 default hydrological parameters on hydrological predictions using 28 error metrics that capture different flow regimes, evapotranspiration (ET) regimes, and extreme conditions.
2. Large-ensemble (~1,300) hydrological CLM5 outputs that account for hydrological parameter uncertainties at each basin.
3. Site-level and regional hydrological parameter sensitivity analysis results that clarify the parametric controls for CLM5 hydrological predictability for 28 error metrics.

The 28 error metrics provide a diagnostic evaluation of how closely the model simulates watershed behavior and support the application of CLM5 in a wide range of studies such as flood and drought prediction, reservoir operation and management, hydrological prediction under anthropogenic influence, etc. For instance, reservoir modelers prioritize capturing monthly flows and annual water balances, while ecosystem modelers generally emphasize the importance of predictions pertaining to seasonal low flow or general low flow regimes. In the error metrics dataset, users can select the metric of interest or a weighted multi-objective metric depending on the application.

Although the datasets are generated at gauged CAMELS basins, the full set of 464 basins are clustered to facilitate regional-scale analysis and extend the results to ungauged basins/grid cells over the CONUS. These datasets intend to offer guidance for future CLM5 hydrological applications, including parameter calibration, by reducing parameter dimensionality, identifying the behavioral values of sensitive parameters, characterizing forcing selection effects, and diagnosing potentially inadequate model structure and parameterization.

## Methods

### CLM5 configuration data

**Fig. 2:**
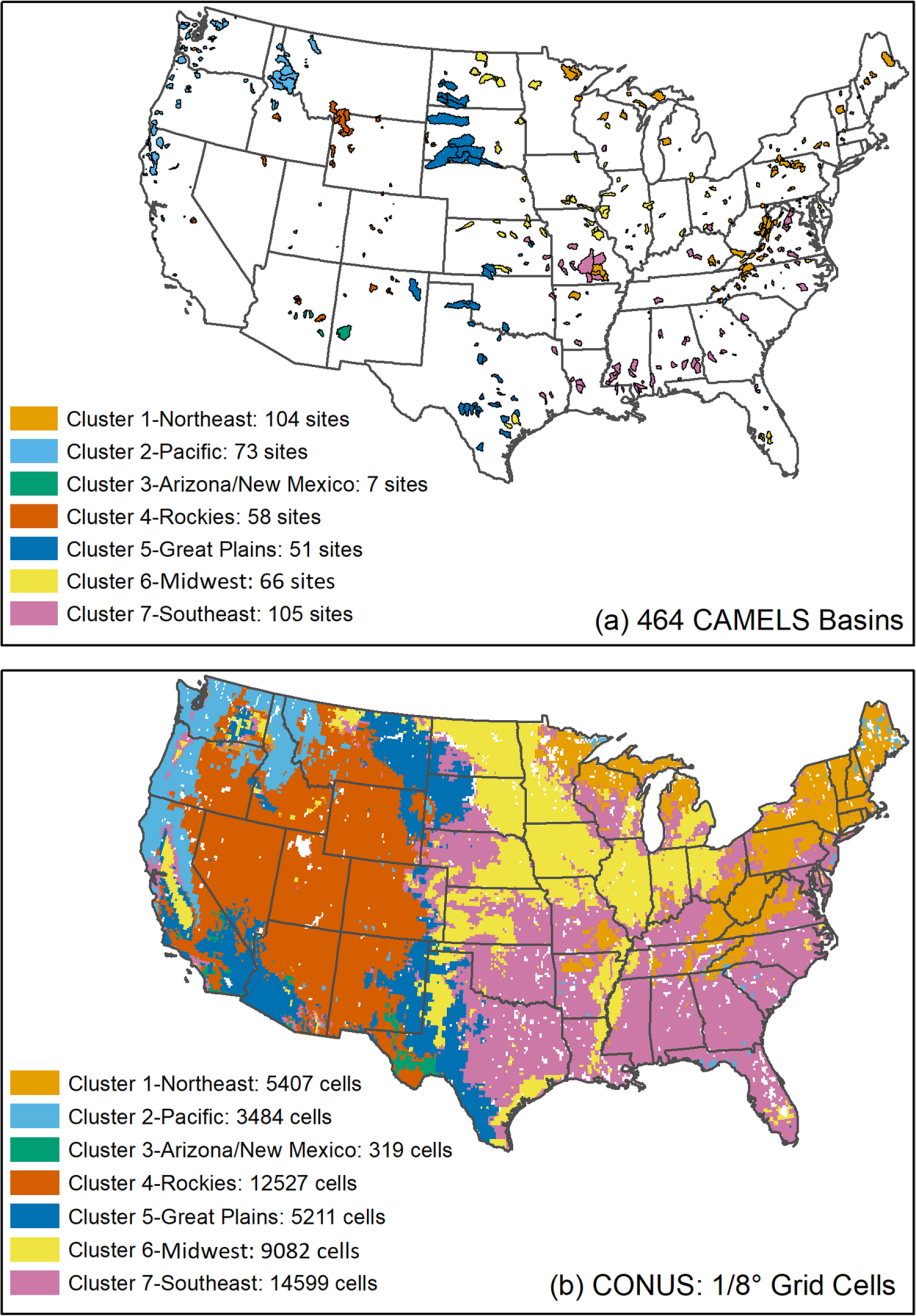
(a) The 464 CAMELS basins and seven clusters defined by the reproducible *k*-means++ algorithm. (b) CONUS 1/8° grid cells placed into the same seven clusters. White areas indicate that lakes and wetland are removed in clustering.

Observational datasets used for CLM5 UC include unregulated daily flow observations for 1980–2014 from the CAMELS dataset, which consists of headwater-type basins with minimal human impacts over the CONUS (Fig. 2a). Monthly ET data at 0.05° grid cell are acquired from the Moderate Resolution Imaging Spectroradiometer (MODIS) products^32^. The basins range in size from about 4 to 25,791 km^2^, with a median basin size of about 436 km^2^. The basin mean elevations range from about 15 m in the Delaware to 3,529 m in the Southern Rocky Mountains, with a median elevation of 458 m.

The five common gridded meteorological forcing datasets include data from Phase 2 of the North American Land Data Assimilation System (NLDAS-2)^33^, Parameter-elevation Regressions on Independent Slopes Model (PRISM)^34^, Daymet^35^, Livneh^36^, and dynamically downscaled European Centre for Medium-Range Weather Forecasts Reanalysis v5^37^ using the Weather Research and Forecasting (WRF-ERA5) model^38^.

Both NLDAS-2 and WRF-ERA5 include hourly precipitation, air temperature, wind speed, surface pressure, specific humidity, and shortwave and longwave radiation data at a 1/8° grid cell over the CONUS. The Livneh data provide daily precipitation, maximum and minimum temperature, and wind speed information at a 1/16° grid cell over the CONUS. Livneh wind speed data are acquired from the National Centers for Enviromental Prediction-National Center for Atmospheric Research (NCEP-NCAR) reanalysis^39^. PRISM and Daymet data provide daily precipitation as well as maximum and minimum temperature information at 4 km and 1 km grid cells over the CONUS, respectively. We use the Mountain Micro Climate Simulator algorithm^40^ to disaggregate daily Livneh, PRISM, and Daymet data into an hourly scale and generate surface pressure, specific humidity, and shortwave and longwave radiation data. Because wind speed data are not provided in PRISM and Daymet data, wind speed is taken from the NLDAS-2 data. The NLDAS-2 data are based on the North American Regional Reanalysis^41^, a major improvement over the earlier NCEP-NCAR reanalysis. All temporal disaggregation is done using the open source Python package MetSim^42^.

The land surface data including land unit type, soil properties, and plant functional type are acquired from the CLM5 input dataset for the CLM5 configuration setting at a 1/8° grid cell over CONUS^13^. The CLM5 land surface data are derived from a variety of sources such as the Moderate Resolution Imaging Spectroradiometer (MODIS) Vegetation Continuous Fields product, the Global Land One-km Base Elevation Project, and the International Geosphere-Biosphere Programme, among others^15^. In addition to the CLM5 land surface data, we also include the 1-km grid cell baseflow index^43^ (upscaled to 1/8° grid cell) over the CONUS for basin clustering. At each CAMELS basin, we estimate the basin mean meteorological forcing, ET, land surface data, and baseflow index from the overlapped grid cells using the area-weighted average method.

### Basin clustering

A total of 22 physical features are selected for each CAMELS basin for clustering (Supplementary Table 1). We classify the 22 features into five categories (topography, land use, soil properties, climate, and other) depending on their function^44^. Several features within each category are highly correlated (i.e., pairs of features that exhibit a Pearson correlation coefficient >0.7). We remove these redundant features and select one representative feature from each correlated group, adding them to independent features that are not strongly correlated with any others. For example, ELEV and STD_ELEV in the “Topography” category are highly correlated, so only ELEV is used in the clustering. SOIL_COLOR is not strongly correlated with other features within the “Soil” category, but is strongly correlated with SLOPE in the “Topography” category. Thus, we did not keep SOIL_COLOR in the clustering analysis. We used a final total of 17 features in the clustering. Note that we do not include streamflow as a clustering criterion. This will allow the clustering analysis to be applied areas of the CONUS where no flow records are available. We use the *k*-means++ clustering^45,46^ with the bootstrapping method to find a stable and reproducible clustering system.

Multiple clusters (cluster size 3 to 10) are tested in the clustering process to identify the optimal number of clusters. First, we randomly partition 90% of the 464 basins as training sets and leave the remaining 10% as validation sets for each cluster number. We then bootstrap 70% of the training sets 40 times and build 40 clustering models. Finally, we classify the validation sets and select the cluster number with highest reproducibility based on four cluster similarity indices: (1) the Rand Index^47^, (2) the Adjusted Rand Index^48^, (3) the Jaccard Index^49^, and (4) the Fowlkes–Mallows Index^50^. Our results suggest that a cluster size of seven has the highest similarity measures for all four indices. Therefore, we use seven clusters for regional analysis (Fig. 2a). Figure 2b shows the 50,629 1/8° grid cells over the CONUS grouped into 7 corresponding clusters.

### CLM5 hydrological parameters

We used the CLM5 Perturbed Parameter Ensembles version, recently developed at NCAR^51^, to perform land surface simulations and produce hydrological datasets. The CLM5-Perturbed Parameter Ensembles configuration allows users to perturb default parameter values. For spatially distributed parameters such as soil porosity and hydraulic conductivity, spatially uniform scaling factors are introduced to preserve the underlying structure. Parameters related to hydrological processes in CLM5 can be classified into six groups: (1) canopy water, (2) surface water, (3) soil water, (4) subsurface water, (5) snow, and (6) evaporation. In this study, we include parameters that cover all six groups in an attempt to gain a comprehensive understanding of the role of CLM5 hydrological parameters in hydrological predictions. Based on previous studies^18–20^ and discussions with CLM5 core developers (i.e., the co-authors D. Kennedy and S. Swenson), we identified 15 hydrological parameters that likely have dominant impacts on the simulation of surface and subsurface runoff, evaporation, canopy water, snow, and soil moisture. Table 1 shows the default parameter values and their prior ranges based on the expert judgement of CLM5 developers.

**Table 1:** The 15 selected hydrological parameters, relevant processes, default values, and prior ranges.

Name	Parameter Definition (unit)	Relevant Hydrological Process	Default Value	Prior Range
fff	Decay factor for fractional saturated area (1/m)	Surface runoff	0.5	[0.02, 5]
N_bf_	Drainage power exponent	Subsurface runoff	1	[1, 2]
K_bf_	Scalar multiplier for base flow rate	Subsurface runoff	0.01	[0.0005, 0.1]
S_y_	Minimum specific yield	Subsurface runoff	0.02	[0.01, 0.02]
B	Scalar multiplier for hydraulic conductivity power exponent	Soil water	1	[0.8, 1.2]
ψ_sat_	Scalar multiplier for saturated soil matric potential	Soil water	1	[0.1, 5]
k_sat_	Scalar multiplier for saturated hydraulic conductivity	Soil water	1	[0.1, 5]
Ѳ_sat_	Scalar multiplier for water content at saturation (porosity)	Soil water	1	[0.8, 1.2]
N_melt_	Parameter controlling shape of snow covered area	Snow	200	[180, 220]
k_acc_	Accumulation constant for fractional snow covered area	Snow	0.1	[0.1, 0.4]
p_sno_	Maximum storage of snow on leaf surface (kg/m^2^)	Canopy water	6	[1.4, 9.5]
p_lip_	Maximum storage of liquid water on leaf surface (kg/m2)	Canopy water	0.1	[0.05, 2]
f_wet_	Maximum fraction of leaf that may be wet prior to the occurrence of dripping	Canopy water	0.05	[0.01, 0.5]
d_max_	Dry surface layer (DSL) parameter (mm)	ET	15	[10, 60]
Ѳ_ini_	Fraction of saturated soil for moisture value at which DSL initiates	ET	0.8	[0.5, 1]

### Ensemble simulation and sensitivity analysis

CLM5 is configured for each basin for ensemble simulation. For each basin, we sample 1,500 parameter sets from their uniform prior distributions using the Latin Hypercube Sampling (LHS) method^52^, which can effectively sample full parameter ranges by dividing the parameter space evenly for representative sample draws. This results in a total of 1,500 × 464 × 5 = 3,480,000 CLM5 simulations. For the default and each ensemble parameter set, we run CLM5 in the satellite phenology mode for 2005–2014. This 10-year simulation period represents the CONUS flooding climatology^53^ and contains extreme hydrological events, which are important for characterizing CLM5 predictability and uncertainty in simulating extreme events. These events include major flooding and droughts such as the 2005 Pacific Northwest drought, the 2012 central Great Plains drought, and the 2012–2016 California exceptional drought. Before the 10-year simulation, each CLM5 run was spun up for 25 years to equilibrate all states^54^. All simulations were performed on the National Energy Research Scientific Computing Center (NERSC) Cori high-performance computing (HPC) system.

Due to parameter interactions that may result in nonphysical states and failed runs, our goal was to obtain at least 1,000 successful CLM5 simulations for each of the 464 CAMELS basins for each forcing dataset. We found that about 10% of the 1,500 parameter sets failed to converge for several basins for each meteorological forcing, resulting in ~1,300 successful CLM5 runs in each basin for the parameter uncertainty characterization and sensitivity analysis for each meteorological forcing. Investigating the runs that failed due to water balance error did not lead to any spatial or parameter-based patterns. All sampled parameters are within their physical ranges, but their complex interactions combined with local climates likely result in nonphysical simulated states and lead to failed runs. Different parameter sets failed in different basins and meteorological forcings, suggesting that parameter interactions vary with the basin and climate. Numerical experiments must be carefully designed to tease out the source of the error and relevant parameters for locations with different climate regimes. However, that work is beyond the scope of this study.

After producing the ensemble simulations, we use the Delta moment-independent sensitivity analysis method (Delta-MIM) to calculate the sensitivity score of the 15 hydrological parameters^55,56^. We selected Delta-MIM for this study because it does not require a specific sampling scheme and includes effects of high-order statistical moments in the response metrics of interest^57^. Delta-MIM exploits an empiric density-based measure that identifies the parameters that most influence the entire distribution of the response variable (i.e., it captures higher order interactive effects beyond mean and variance responses). For each parameter, the resulting Delta index measures the normalized expected shift in the distribution of the response variable induced by the parameter.

### Diagnostic error metrics

We include a total of 28 error metrics to comprehensively assess CLM5 performance, uncertainty, hydrological parameter sensitivity to different flow regimes (e.g., high/low flows, water balance, etc.), and ET characteristics at different temporal scales (e.g., seasonal and annual). Table 2 presents these metrics. Their relevant scales and mathematical descriptions are provided in the Supplementary Information.

**Table 2:** Description of the 28 error metrics.

Variable	Error Metric	Unit	Relevance
Flow	Daily Kling Gupta Efficiency (KGE)	—	Multiobjective metric
	Daily Mean Absolute Error (MAE)	m^3^/s	Overall daily flow
	Daily Nash Sutcliffe Efficiency (NSE)	—	High daily flow
	Daily Root Mean Square Error (RMSE)	m^3^/s	High daily flow
	Daily Transformed Root Mean Square Error (TRMSE)	m^3^/s	Low daily flow
	Daily Variance Bias	—	Daily flow variability
	Monthly KGE	—	Multiobjective metric
	Monthly MAE	m^3^/s	Overall monthly flow
	Monthly NSE	—	High monthly flow
	Monthly RMSE	m^3^/s	High monthly flow
	Monthly TRMSE	m^3^/s	Low monthly flow
	Monthly Variance Bias	—	Monthly flow variability
	Annual Volume Bias	—	Total water balance
	Flow Regime Quantile 0–10% (Q0–10) Volume Bias	—	Low flow water balance
	Q10–25 Volume Bias	—	Low flow water balance
	Q25–50 Volume Bias	—	Moderate flow water balance
	Q50–75 Volume Bias	—	Moderate flow water balance
	Q75–90 Volume Bias	—	High flow water balance
	Q90–100 Volume Bias	—	High flow water balance
	Winter (DJF) Volume Bias	—	Seasonal water balance
	Spring (MAM) Volume Bias	—	Seasonal water balance
	Summer (JJA) Volume Bias	—	Seasonal water balance
	Fall (SON) Volume Bias	—	Seasonal water balance
ET	Annual Bias	—	Total water balance
	Winter (DJF) Bias	—	Seasonal water balance
	Spring (MAM) Bias	—	Seasonal water balance
	Summer (JJA) Bias	—	Seasonal water balance
	Fall (SON) Bias	—	Seasonal water balance

## Data Records

The CLM5 hydrological datasets are publicly available in comma-separated value (.csv) and netcdf (.nc) formats and hosted in the MultiSector Dynamics – Living, Intuitive, Value-adding, Environment (MSD-LIVE) data repository^58^. Due to page limitation, Table 3 only provides an example of the data structures, data files, and variables. Full data descriptions can be found in the README file in the repository.

**Table 3:** Description of the CLM5 hydrological datasets.

Main Folder	File Naming Convention & Description	Data Description*
	*1500_ensemble_parameters.csv* Description of 1,500 parameter sets	**Data Dimension**: 1,501 (R) × 16 (C). **C1:** Parameter set ID; **C2‒C16:** 15 hydrological parameters in the same order as listed in Table 1
	*Features_CAMELS_basins.csv* Feature values of 464 CAMELS basins used for clustering	**Data Dimension**: 465 (R) × 27 (C). **C1:** Number; **C2:** Basin ID; **C3:** Latitude; **C4:** Longitude; **C5:** Cluster ID; **C6‒C27:** 22 feature as listed in Supplementary Table 1
	*Features_CONUS_cells.csv* Feature values of 50,629 1/8° grid cells over the CONUS	**Data Dimension**: 50,630 (R) × 26 (C). **C1:** Number; **C2:** Latitude; **C3:** Longitude; **C4:** Cluster ID; **C5‒C26:** 22 feature as listed in Supplementary Table 1
[Met]_forcing/e.g., NLDAS2_forcing/	*Parameter_id.csv* ~1,300 parameter set ID that successfully finish CLM5 runs for all 464 basins	**Data Dimension**: ~1,300 (R) × 1 (C). **C1:** Successful parameter set ID, consistent with the *1500_ensemble_parameters.csv*
[Met]_forcing/Flow_series_default_parameter	Daily streamflow (in m^3^/s) time series of CLM5 simulations from 2005‒2014 using default parameters (extracted from the netcdf files) for 464 CAMELS basins	
	*[basin ID_daily].csv* e.g., *01030500_daily.csv*	**Data Dimension**: 3,651 (R) × 5 (C). **C1‒C3:** dates (year, month, day); **C4:** CLM5 flow; **C5:** observed flow from CAMELS datasets
[Met]_forcing/Flow_series_ensemble_parameter	Ensemble daily streamflow (in m^3^/s) time series of CLM5 simulations from 2005‒2014 using ~1,300 parameter sets (extracted from the netcdf files) for 464 CAMELS basins	
	*[basin ID_daily_ensemble].csv* e.g., *01030500_daily_ensemble.csv*	**Data Dimension**: 3,651 (R) × ~1,300 (C). C1‒C~1300: CLM5 daily flow using the ensemble parameters. Consecutive number is used in the column which is associated with parameter ID in *Parameter_id.csv*
[Met]_forcing/Flow_ET_metrics	28 error metric values for the default parameter run and ~1,300 ensemble parameter runs for the 464 CAMELS basins. Units are shown in the *CLM5_default_parameter_28_metrics.csv*	
	*CLM5_default_parameter_28_metrics.csv*	Data Dimension: 465 (R) × 33 (C). **C1:** Number; **C2:** Basin ID; **C3:** Latitude; **C4:** Longitude; **C5:** Cluster ID; **C6‒C33:** values of 28 error metrics as listed in Table 2
	*[CLM5_ensemble_metric].csv* e.g., *CLM5_ensemble_daily_KGE.csv*	**Data Dimension**: 465 (R) × ~1,300 (C). **C1:** Basin ID; **C2:** Latitude; **C3:** Longitude; **C4:** Cluster ID; C5‒C~1300: error metric values for ensemble parameters. Consecutive number is used in the column which is associated with parameter ID in *Parameter_id.csv*
[Met]_forcing/Sensitivity_scores	Normalized sensitivity score [0‒1] using the Delta moment-independent method for 28 error metrics at 464 CAMELS basins	
	*[delta_metric].csv* e.g., *delta_daily_KGE.csv*	**Data Dimension**: 465 (R) × 19 (C). **C1:** Basin ID; **C2:** Latitude; **C3:** Longitude; **C4:** Cluster ID; **C5‒C19:** Normalized sensitivity score for 15 hydrological parameters in the same order as listed in Table 1

*Note: In “Data Description”, C = column, R = Row. C[i] indicates the ith column of a data file.

## Technical Validation

**Fig. 3:**
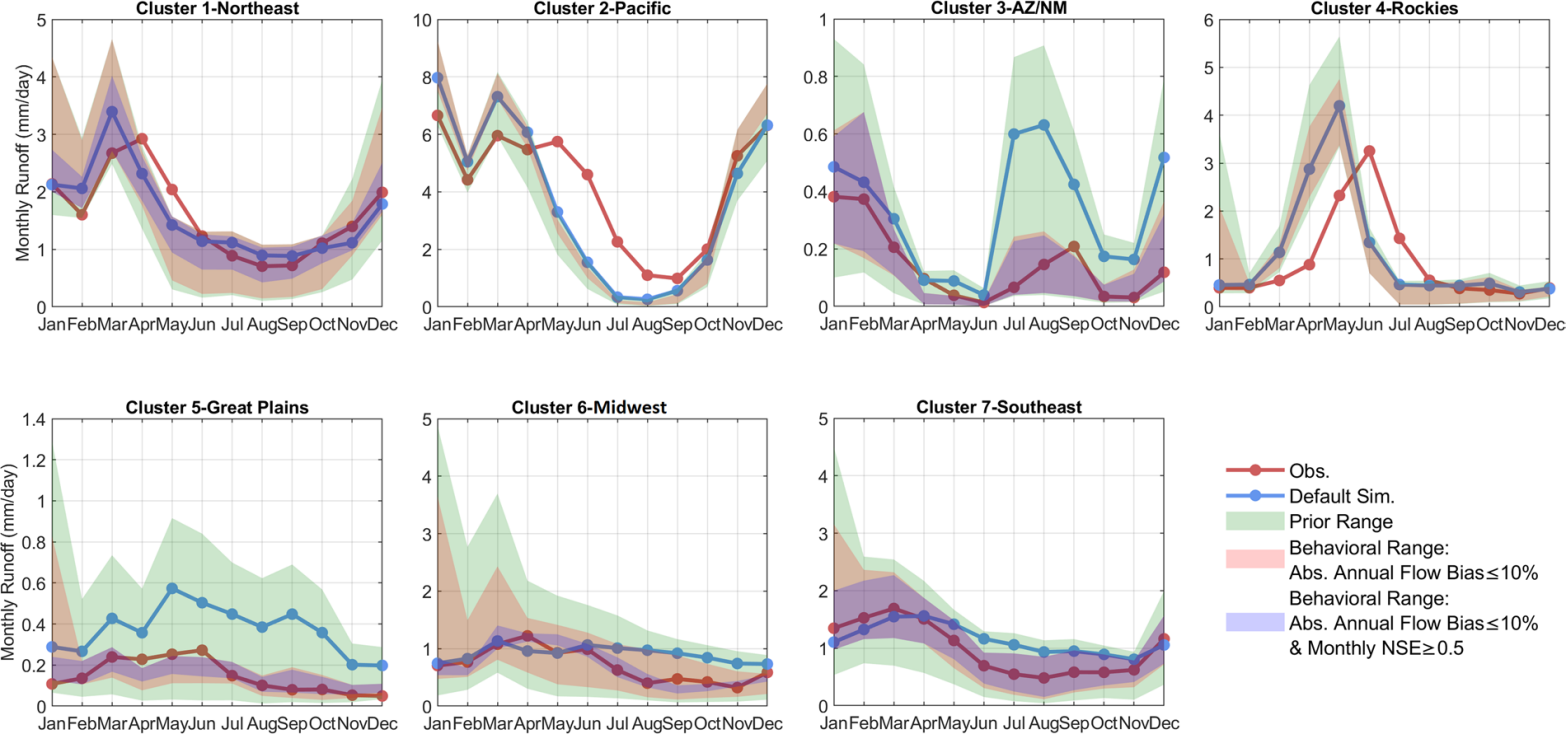
Regional mean monthly flow using the NLDAS-2 forcing data in the 7 clusters. The green spread indicates all ~1,300 ensemble members. The red shading indicates the spread for parameter sets that have annual flow bias within 10% of the observed flows. The blue shading indicates the spread for parameter sets that have annual flow bias within 10% of the observed flows and an NSE value of monthly flow above or equal to 0.5.

The accuracy and precision of the CLM5 ensemble streamflow simulations depend on partitioning the “behavioral” and “nonbehavioral” parameter sets using streamflow measurements, which differ for each error metric and threshold value. Simulations that produce error metrics that fall within user-defined acceptable performance metric ranges are considered “behavioral”, while those that fall outside these ranges are “non-behavioral”. In the following discussion, we use CLM5 ensemble simulations driven by the NLDAS-2 meteorological forcing data as an example and perform similar analyses for the other meteorological forcing datasets. Figure 3 shows the spread of regional monthly runoff in 7 clusters using two different constraints to partition behavioral parameter sets: (1) annual flow bias within 10% and (2) annual flow bias within 10% and monthly NSE higher than 0.5. Despite biases in a few regions (i.e., underestimating the summer flow in Cluster 2-Pacific and a flow peak time mismatch in Cluster 4-Rockies), the behavioral ensemble simulations that satisfy either constraint significantly improve default parameter simulation for all clusters and better reproduce observed flow. Using the single best performing set based on the monthly KGE metric, CLM5 skill for simulating monthly streamflow in 2005–2014 can be improved from 0.8586 with the default parameters to 0.8637 in Cluster 1-Northeast, from 0.6476 to 0.7278 in Cluster 2-Pacific, from ‒0.3448 to 0.9110 in Cluster 3-AZ/NM, from 0.4089 to 0.4750 in Cluster 4-Rockies, from ‒0.5674 to 0.8624 in Cluster 5-Great Plains, from 0.2836 to 0.7974 in Cluster 6-Midwest, and from 0.6004 to 0.9233 in Cluster 7-Southeast.

## Usage Notes

The CLM5 hydrological datasets listed in Table 3 can be directly used for a wide variety of applications over different spatial scales ranging from local, to regional, to the full CONUS. We present the major three data usage applications here, but our choices are not exhaustive.

1. *Characterize meteorological and hydrological parameter uncertainty*. For each meteorological forcing, the ~1,300 hydrological parameter sets and their ensemble simulations can be directly used to study the impacts of hydrological parameter uncertainty on hydrological predictions. One notable example is assessing the relative role of parameter uncertainty and choice of meteorological forcing in simulating different flow regimes. For projection studies, users also can assess the relative roles of hydrological parameter uncertainty and climate or land use change uncertainty on future hydrological changes. At the CAMELS basin scale, users can directly employ the ensemble streamflow prediction datasets to characterize uncertainty. For ungauged basins in the CONUS, users can find the basin cluster as shown in Fig. 2b and then approximate parameter uncertainty with the spread of regional streamflow as shown in Fig. 3.
2. *Guide hydrological parameter calibration (deterministic) and behavioral parameter selection (ensemble) at both CAMELS basins and ungauged basins*. In practice, the accuracy and precision of the CLM5 ensemble streamflow simulations depend on the partitioning of behavioral and nonbehavioral parameter sets. Simulations that produce error metrics that fall within user-defined acceptable performance metric ranges (e.g., NSE ≥ 0.5 in Fig. 3) are considered behavioral, while those that fall outside these ranges are non-behavioral. Figure 4 shows the sensitivity scores of the 464 basins to the annual flow bias metric and the regional sensitivity scores to 28 error metrics for Cluster 1-Northeast, using NLDAS-2 forcing data as an example. These results can aid in future CLM5 hydrological parameter calibration efforts by reducing parameter dimensionality with sensitive parameters and identifying their behavioral values for different error metrics. At the CAMELS basin scale, users can directly select the best performance parameter set for their metric of interest (such as seasonal or annual flow bias for reservoir modeling) to perform deterministic simulations or select ensemble behavioral parameter sets with one or more metric constraints. At ungauged basins, users first identify their basin cluster number. They then use the regional sensitivity score such as Fig. 4b to identify the sensitive parameters and find their behavioral parameter values. The sensitivity scores for the 28 error metrics can support a wide range of hydrological applications.

**Fig. 4:**
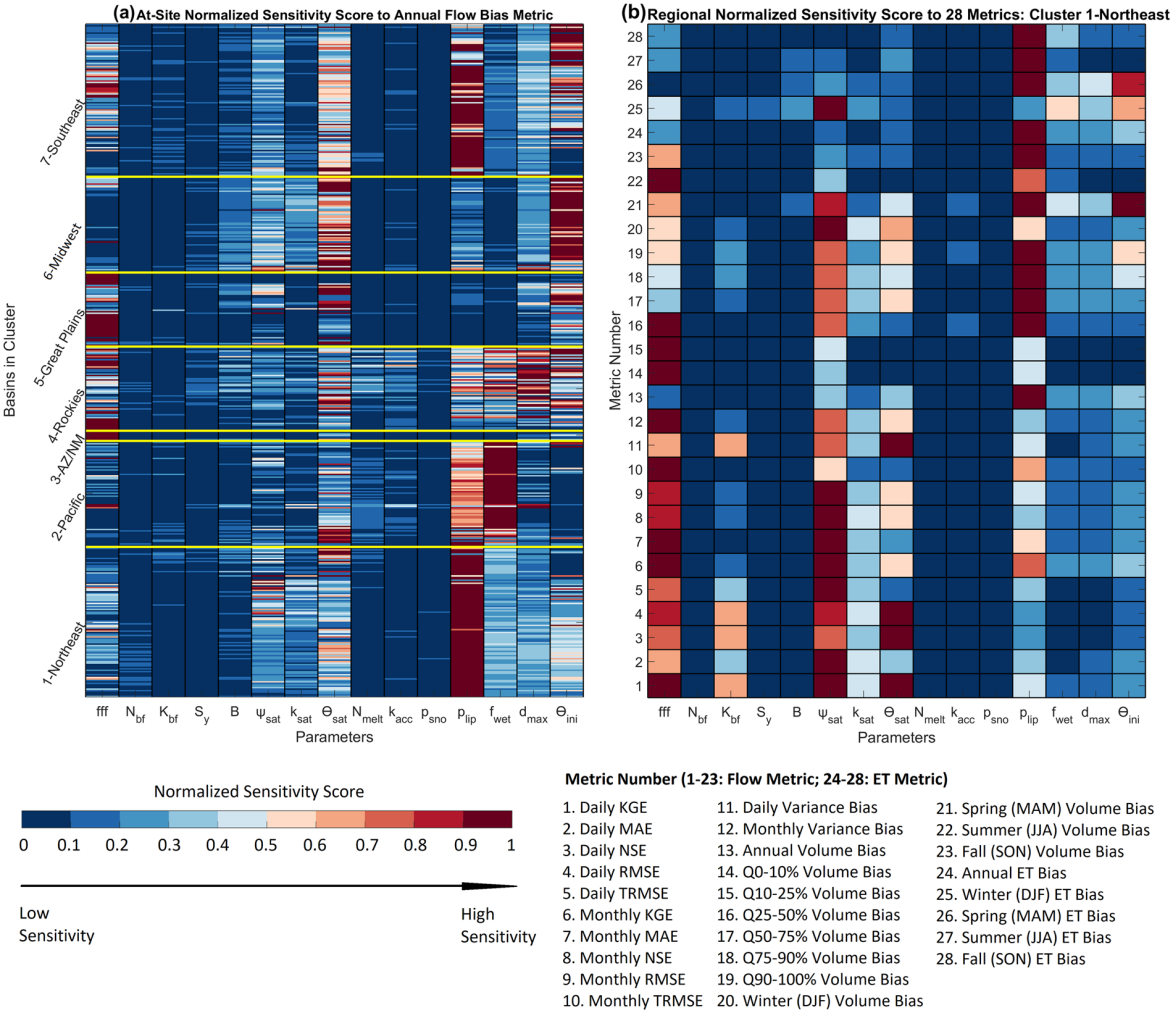
(**a**) The normalized sensitivity score of the 15 hydrological parameters to the annual flow bias metric at each basin in each cluster. (**b**) Regional normalized sensitivity score to 28 diagnostic error metrics using Cluster 1-Northeast and NLDAS-2 forcing data as an example.
3. *Aid CLM5 model developers in diagnosing potentially inadequate model structures and parameterizations*. For example, Fig. 3 shows that no parameter set meets the constraint that monthly flow NSE is higher than 0.5 in Cluster 4-Rockies using the NLDAS-2 forcing data. This indicates very poor performance and some errors in model structure for high flow simulation and timing in this region. The earlier peak flow may be related to CLM5’s lack of representation of sub-grid topographic variability and how it impacts solar radiation, which is critical to correctly timing snow melt. The small value in the depth-to-bedrock parametrization for Cluster 2-Pacific (i.e., mean value of 1.08 m) may help explain the underestimation of summer low flow due to the predicted low soil water-holding capacity.

Note that CAMELS basins are small to mid-size basins with minimal human intervention. For users who are interested in modeling the large river systems typically influenced by human activities such as reservoir operations, these data sets can produce enhanced CLM5 runoff simulations as input for downstream river routing and water management models^59,60^.

## Supplementary information

Supporting Information

## Data Availability

The CLM5 hydrological datasets are available to the public at 10.57931/1922953 in comma-separated value (.csv) and netcdf (.nc) formats. This experiment used a modified version of CLM5 designed to allow easier parameterization and support machine-specific compilation. The modified source code is available at 10.5281/zenodo.6653704^61^, forked from https://github.com/ESCOMP/CTSM/tree/branch_tags/PPE.n11_ctsm5.1.dev030. Source codes that were used to develop and analyze the data are available at 10.5281/zenodo.7039118^62^. The MetSim disaggregation code is available at https://github.com/UW-Hydro/MetSim.

## References

[1] Bales RC (2006). Mountain hydrology of the western United States. Water Resour. Res..

[2] Barnett TP, Adam JC, Lettenmaier DP (2005). Potential impacts of a warming climate on water availability in snow-dominated regions. Nature.

[3] Yan H, Sun N, Chen X, Wigmosta MS (2020). Next-Generation Intensity-Duration-Frequency Curves for Climate-Resilient Infrastructure Design: Advances and Opportunities. Front. Water.

[4] Yan H (2019). Observed Spatiotemporal Changes in the Mechanisms of Extreme Water Available for Runoff in the Western United States. Geophys. Res. Lett..

[5] Hou, Z. *et al*. Incorporating climate nonstationarity and snowmelt processes in intensity–duration–frequency analyses with case studies in mountainous areas. *J. Hydrometeorol*. **20** (2019).

[6] Yan H (2018). Next-Generation Intensity-Duration-Frequency Curves for Hydrologic Design in Snow-Dominated Environments. Water Resour. Res..

[7] Sun N (2022). Datasets for characterizing extreme events relevant to hydrologic design over the conterminous United States. Sci. Data.

[8] Turner SWD, Hejazi M, Kim SH, Clarke L, Edmonds J (2017). Climate impacts on hydropower and consequences for global electricity supply investment needs. Energy.

[9] Yan H, Sun N, Fullerton A, Baerwalde M (2021). Greater vulnerability of snowmelt-fed river thermal regimes to a warming climate. Environ. Res. Lett..

[10] Zarekarizi, M., Yan, H., Ahmadalipour, A. & Moradkhani, H. A Probabilistic Framework for Agricultural Drought Forecasting Using the Ensemble Data Assimilation and Bayesian Multivariate Modeling. in *Global Drought and Flood: Observation*, *Modeling, and Prediction* 147–164, 10.1002/9781119427339.ch8 (2021).

[11] Cheng Y (2021). Validation of the Community Land Model Version 5 Over the Contiguous United States (CONUS) Using *In Situ* and Remote Sensing Data Sets. J. Geophys. Res. Atmos..

[12] Swenson SC, Lawrence DM (2014). Assessing a dry surface layer-based soil resistance parameterization for the Community Land Model using GRACE and FLUXNET-MTE data. J. Geophys. Res. Atmos..

[13] Lawrence D (2019). The Community Land Model Version 5: Description of New Features, Benchmarking, and Impact of Forcing Uncertainty. J. Adv. Model. Earth Syst..

[14] Li H-Y (2015). Evaluating Global Streamflow Simulations by a Physically Based Routing Model Coupled with the Community Land Model. J. Hydrometeorol..

[15] Lawrence, D. *et al*. *CLM5 Documentation*. (2020).

[16] Cherchi A (2019). Global mean climate and main patterns of variability in the CMCC‐CM2 coupled model. J. Adv. Model. Earth Syst..

[17] Bentsen M (2013). The Norwegian Earth System Model, NorESM1-M – Part 1: Description and basic evaluation of the physical climate. Geosci. Model Dev..

[18] Ren H (2016). Classification of hydrological parameter sensitivity and evaluation of parameter transferability across 431 US MOPEX basins. J. Hydrol..

[19] Huang M (2013). Uncertainty Analysis of Runoff Simulations and Parameter Identifiability in the Community Land Model: Evidence from MOPEX Basins. J. Hydrometeorol..

[20] Hou Z, Huang M, Leung LR, Lin G, Ricciuto DM (2012). Sensitivity of surface flux simulations to hydrologic parameters based on an uncertainty quantification framework applied to the Community Land Model. J. Geophys. Res. Atmos..

[21] Mendoza PA (2015). Are we unnecessarily constraining the agility of complex process-based models?. Water Resour. Res..

[22] Pelletier JD (2016). A gridded global data set of soil, intact regolith, and sedimentary deposit thicknesses for regional and global land surface modeling. J. Adv. Model. Earth Syst..

[23] Gou J (2020). Sensitivity Analysis‐Based Automatic Parameter Calibration of the VIC Model for Streamflow Simulations Over China. Water Resour. Res..

[24] Zarekarizi M, Srikrishnan V, Keller K (2020). Neglecting uncertainties biases house-elevation decisions to manage riverine flood risks. Nat. Commun..

[25] Karimi, T., Reed, P., Malek, K. & Adam, J. Diagnostic Framework for Evaluating How Parametric Uncertainty Influences Agro‐Hydrologic Model Projections of Crop Yields Under Climate Change. *Water Resour. Res*. **58** (2022).

[26] Reed PM (2022). Multisector Dynamics: Advancing the Science of Complex Adaptive Human‐Earth Systems. Earth’s Futur..

[27] Reed, P. M. *et al*. *Addressing Uncertainty in Multisector Dynamics Research*. (Zenodo, 2022).

[28] Addor N, Newman AJ, Mizukami N, Clark MP (2017). The CAMELS data set: catchment attributes and meteorology for large-sample studies. Hydrol. Earth Syst. Sci..

[29] Newman AJ (2015). Development of a large-sample watershed-scale hydrometeorological data set for the contiguous USA: data set characteristics and assessment of regional variability in hydrologic model performance. Hydrol. Earth Syst. Sci..

[30] Viger RJ, Bock A (2014). GIS Features of the Geospatial Fabric for National Hydrologic Modeling..

[31] Falcone, J. A. GAGES-II: Geospatial Attributes of Gages for Evaluating Streamflow, Digital spatial data set 2011, available at: http://water.usgs.gov/GIS/metadata/usgswrd/XML/gagesII_Sept2011.xml (2011).

[32] Mu Q, Zhao M, Running SW (2011). Improvements to a MODIS global terrestrial evapotranspiration algorithm. Remote Sens. Environ..

[33] Xia Y (2012). Continental-scale water and energy flux analysis and validation for the North American Land Data Assimilation System project phase 2 (NLDAS-2): 1. Intercomparison and application of model products. J. Geophys. Res. Atmos..

[34] Daly C, Neilson RP, Phillips DL (1994). A Statistical-Topographic Model for Mapping Climatological Precipitation over Mountainous Terrain. J. Appl. Meteorol..

[35] Thornton PE, Running SW, White MA (1997). Generating surfaces of daily meteorological variables over large regions of complex terrain. J. Hydrol..

[36] Livneh B (2013). A Long-Term Hydrologically Based Dataset of Land Surface Fluxes and States for the Conterminous United States: Update and Extensions. J. Clim..

[37] Hersbach H (2020). The ERA5 global reanalysis. Q. J. R. Meteorol. Soc..

[38] Jones AD (2022). IM3/HyperFACETS Thermodynamic Global Warming (TGW) Simulation Datasets (v1.0.0) [Dataset]. MSD-LIVE Data Repository..

[39] Kalnay E (1996). The NCEP/NCAR 40-Year Reanalysis Project. Bull. Am. Meteorol. Soc..

[40] Thornton PE, Running SW (1999). An improved algorithm for estimating incident daily solar radiation from measurements of temperature, humidity, and precipitation. Agric. For. Meteorol..

[41] Mesinger F (2006). North American Regional Reanalysis. Bull. Am. Meteorol. Soc..

[42] Bennett A, Hamman J, Nijssen B (2020). MetSim: A Python package for estimation and disaggregation of meteorological data. J. Open Source Softw..

[43] Wolock, D. M. *Base-flow index grid for the conterminous United States*. (2003).

[44] Yadav M, Wagener T, Gupta H (2007). Regionalization of constraints on expected watershed response behavior for improved predictions in ungauged basins. Adv. Water Resour..

[45] Arthur, D. & Vassilvitskii, S. k-means++: The advantages of careful seeding. in *Proc. of the 18th annual ACM-SIAM symposium on discrete algorithms* 1027–1035 (2007).

[46] von Luxburg U (2010). Clustering Stability: An Overview. Found. Trends Mach. Learn..

[47] Rand WM (1971). Objective Criteria for the Evaluation of Clustering Methods. J. Am. Stat. Assoc..

[48] Hubert L, Arabie P (1985). Comparing partitions. J. Classif..

[49] Halkidi M, Batistakis Y, Vazirgiannis M (2001). On Clustering Validation Techniques. J. Intell. Inf. Syst..

[50] Fowlkes EB, Mallows CL (1983). A Method for Comparing Two Hierarchical Clusterings. J. Am. Stat. Assoc..

[51] Community Terrestrial Systems Model (includes the Community Land Model of CESM). https://github.com/ESCOMP/CTSM/tree/branch_tags/PPE.n11_ctsm5.1.dev030 (2022).

[52] Mckay MD, Beckman RJ, Conover WJ (2000). A Comparison of Three Methods for Selecting Values of Input Variables in the Analysis of Output From a Computer Code. Technometrics.

[53] Dougherty E, Rasmussen KL (2019). Climatology of Flood-Producing Storms and Their Associated Rainfall Characteristics in the United States. Mon. Weather Rev..

[54] Dagon K, Sanderson BM, Fisher RA, Lawrence DM (2020). A machine learning approach to emulation and biophysical parameter estimation with the Community Land Model, version 5. Adv. Stat. Climatol. Meteorol. Oceanogr..

[55] Borgonovo E (2007). A new uncertainty importance measure. Reliab. Eng. Syst. Saf..

[56] Plischke E, Borgonovo E, Smith CL (2013). Global sensitivity measures from given data. Eur. J. Oper. Res..

[57] Hadjimichael A, Quinn J, Reed P (2020). Advancing Diagnostic Model Evaluation to Better Understand Water Shortage Mechanisms in Institutionally Complex River Basins. Water Resour. Res..

[58] Yan H (2023). CLM5 CAMELS Basins Ensemble (v1.0.0) [Dataset]. MSD-LIVE Data Repository..

[59] Turner SWD, Doering K, Voisin N (2020). Data‐Driven Reservoir Simulation in a Large‐Scale Hydrological and Water Resource Model. Water Resour. Res..

[60] Thurber T (2021). mosartwmpy: A Python implementation of the MOSART-WM coupled hydrologic routing and water management model. J. Open Source Softw..

[61] Koch J (2022). Zenodo.

[62] Thurber T (2022). Zenodo.

