# Supplementary material for: Characterizing uncertainty in Community Land Model version 5 hydrological applications in the United States
Source: Sci Data. 2023 Apr 6;10:187. doi: 10.1038/s41597-023-02049-7 (PMC10079652; doi:10.1038/s41597-023-02049-7)
Supplement: Supplementary file 1 — Supporting Information [file 41597_2023_2049_MOESM1_ESM.docx]

Supporting Information for

**Characterizing uncertainty in Community Land Model version 5 hydrological applications in the United States**

Hongxiang Yan^1*^, Ning Sun^1^, Hisham Eldardiry^1^, Travis Thurber^1^, Patrick M. Reed^2^, Keyvan Malek^2^, Rohini Gupta^2^, Daniel Kennedy^3^, Sean C. Swenson^3^, Linying Wang^4^, Dan Li^4^, Chris R. Vernon^1^, Casey D. Burleyson^1^, and Jennie S. Rice^1^

1. Pacific Northwest National Laboratory, Richland, WA, USA

2. Department of Civil and Environmental Engineering, Cornell University, Ithaca, NY, USA

3. National Center for Atmospheric Research, Boulder, CO, USA

4. Department of Earth and Environment, Boston University, MA, USA

Corresponding author: Hongxiang Yan ([hongxiang.yan@pnnl.gov](mailto:hongxiang.yan@pnnl.gov))

**Table of Contents**

Table S1. Features for CAMELS basin and CONUS grid cell clustering…………………………………………………………2

Error Metric Descriptions…………………………………………………………………………………………………………………………3

Reference………………………………………………………………………………………………………………………………………………..4

**Table S1.** Features for CAMELS basin and CONUS grid cell clustering. Red text indicates features that are strongly correlated (r>0.7) with others. They are removed for clustering.

| **Feature** | **Unit** | **Category** | **Description [Data Source]** |
| --- | --- | --- | --- |
| ELEV | m | Topography | Mean elevation [CLM5] |
| STD_ELEV | m |  | Standard deviation of elevation [CLM5] |
| SLOPE | ° |  | Mean topographic slope [CLM5] |
| SAND | % | Soil | Percent sand [CLM5] |
| CLAY | % |  | Percent clay [CLM5] |
| SOIL_DEP | m |  | Soil depth [CLM5] |
| SOIL_COLOR | - |  | Soil color [CLM5] |
| CROP | % | Land Use | Percent crop [CLM5] |
| URBAN | % |  | Percent urban [CLM5] |
| ARIDITY | - | Climate | Mean annual potential evapotranspiration/precipitation [NLDAS2] |
| P_CV | - |  | Coefficient of variation in annual precipitation [NLDAS2] |
| P_Avg | mm |  | Mean annual precipitation [NLDAS2] |
| P_MED_1D | mm |  | Median annual maximum 1-day precipitation [NLDAS2] |
| P_MED_2D | mm |  | Median annual maximum 2-day precipitation [NLDAS2] |
| P_MED_1H | mm |  | Median annual maximum 1-hour precipitation [NLDAS2] |
| SH_Avg | kg/kg |  | Mean annual specific humidity [NLDAS2] |
| B | - | Other | Baseflow ratio [USGS] |
| GRASS | % |  | Percent grass [CLM5] |
| SHRUB | % |  | Percent shrub [CLM5] |
| DECIDUOUS | % |  | Percent deciduous tree [CLM5] |
| GREEN | % |  | Percent evergreen tree [CLM5] |
| BARE | % |  | Percent bare soil [CLM5] |

# Error Metric Descriptions

## **Kling-Gupta efficiency (KGE)**

$KGE=1-\sqrt{{(\beta-1)}^{2}+{(\alpha-1)}^{2}+{(\rho-1)}^{2}}$ (1)

where $\beta={\mu_{s}}/{\mu_{o}}$, $\alpha={\sigma_{s}}/{\sigma_{o}}$, $\mu_{s}$, and $\mu_{o}$ denote means of flow simulation and observation in a given time window; $\sigma_{s}$ and $\sigma_{o}$ denote standard deviation of flow simulation and observation given a time window; and $\rho$ is the Pearson correlation between flow simulation and observation.

## **Nash–Sutcliffe efficiency (NSE)**

$NSE=1-\frac{MSE}{{\sigma_{o}}^{2}}$ (2)

where $MSE$ denotes mean squared error, and $MSE=\frac{1}{N}\sum_{t=1}^{N} {(q_{s}^{t}-q_{o}^{t})}^{2}$, $q_{s}^{t}$ and $q_{o}^{t}$ are the flow simulation and observation at time step $t$ and $N$ is the number of time steps.

## **Mean Absolute Error (MAE)**

$MAE=\frac{\sum_{t=1}^{N} \left| q_{s}^{t}-q_{o}^{t} \right|}{N}$ (3)

## **Root-Mean-Square-Error (RMSE)**

$RMSE=\sqrt{\frac{1}{N}\sum_{t=1}^{N} {(q_{s}^{t}-q_{o}^{t})}^{2}}$ (4)

## **Transformed Root-Mean-Square-Error (TRMSE)**

$TRMSE=\sqrt{\frac{1}{N}\sum_{t=1}^{N} {(\hat{q}_{s}^{t}-\hat{q}_{o}^{t})}^{2}}$ (5)

where $\hat{q}$ is the Box-Cox transformed flow simulation and $\hat{q}=\frac{{(1+q)}^{\lambda}-1}{\lambda}$, $\lambda=0.3$ following Kollat et al. (2012).

## **Flow Variance Bias**

$variance bias= \frac{\sigma_{s}-\sigma_{o}}{\sigma_{o}}\times100$ (6)

## **Flow Volume Bias or Evapotranspiration (ET) Bias**

$volume bias= \frac{\sum_{T} q_{s}^{t}-\sum_{T} q_{o}^{t}}{\sum_{T} q_{o}^{t}}\times100$ (7)

where $T$ indicates the time window. For annual volume bias, $T$ indicates all time steps during a 2005–2014 simulation period; for seasonal volume bias, $T$ indicates all time steps in that season during 2005–2014; for flow regime bias such as Q0-10, $T$ indicates all time steps in the flow regime (estimating using the FDC) from 2005–2014. For ET bias, $q$ indicates ET simulations.

# References

Kollat, J. B., Reed, P. M., & Wagener, T. (2012). When are multiobjective calibration trade‐offs in hydrologic models meaningful?. Water Resources Research, 48(3).
